# The Ether Discovery

**Published:** 1851-11

**Authors:** 


THE ETHER DISCOVERY.

A very severe and to some extent a bitter controversy sprung up sometime ago, on the relative merits of Dr. C. T. Jackson and Dr. Morton in the discovery of etherization. A great deal of testimony was collected by the parties, and the general impression has settled down in favor of the claims of Dr. Jackson. The Academy of Sciences, of France, awarded to him the honor, and Dr. Charles Lyell has recently written a letter, in which, after a thorough examination of the testimony, he gives the credit of priority in the discovery, to Dr. Jackson.

